# The Impact of Racial and Non-racial Discrimination on Health Behavior Change Among Visible Minority Adults During the COVID-19 Pandemic

**DOI:** 10.1007/s40615-021-01189-z

**Published:** 2021-11-29

**Authors:** Cheryl L. Currie, Erin K. Higa

**Affiliations:** Faculty of Health Sciences, University of Lethbridge, M3083 Markin Hall, 4401 University Drive, Lethbridge, AB T1K 3M4 Canada

**Keywords:** Discrimination, Ethnic/racial, Alcohol, Sleep, Exercise, Behavior, COVID-19

## Abstract

**Introduction:**

Pre-pandemic health behavior has been put forward as a reason for excess COVID-19 infection and death in some racialized groups. At the same time, scholars have labeled racism the other pandemic and argued for its role in the adverse COVID-19 outcomes observed. The purpose of this study was to examine the impact of discrimination on health behavior change among racialized adults in the early stages of the pandemic.

**Methods:**

Data were collected from 210 adults who identified as a visible minority in Alberta, Canada, in June 2020. The Everyday Discrimination Scale (Short Version) was adapted to examine past-month experiences. Four questions asked if alcohol/cannabis use and stress eating had significantly increased, and if sleep and exercise had significantly decreased in the past month. Logistic regression models examined associations between discrimination attributed to racial and non-racial causes and health behavior change adjusted for covariates.

**Results:**

The majority of adults (56.2%) reported past-month discrimination including 26.7% who attributed it to their race. Asian adults reported more racial discrimination and discrimination due to people believing they had COVID-19 than other visible minorities. Racial discrimination during the pandemic was strongly associated with increased substance use (OR: 4.0, 95% CI 1.2, 13.4) and decreased sleep (OR: 7.0, 95% CI 2.7, 18.4), and weakly associated with decreased exercise (OR: 2.2, 95% CI 1.1, 4.5). Non-racial discrimination was strongly associated with decreased sleep (OR: 4.8, 95% CI 1.8, 12.5).

**Conclusion:**

Racial discrimination may have a particularly important effect on intensifying adverse health behavior changes among racialized adults during a time of global crisis.

## Introduction

Many adults have reported adverse health behavior change during the COVID-19 pandemic including increased substance use, decreased physical activity, and changes in sleep and eating patterns [1–4]. The purpose of this study was to examine the role that racial discrimination may have played in exacerbating health behavior change among visible minorities in the early stages of the pandemic. In Canada, visible minorities are persons, other than Indigenous peoples, who are non-Caucasian in race or non-white in color [5]. Many visible minorities in Canada experience racial discrimination defined as any exclusion, restriction or preference based on race, color, descent, or ethnic origin that has the purpose of impairing the enjoyment of human rights and fundamental freedoms in public life [6–8].

Visible minority groups have often faced discrimination during epidemics and pandemics, particularly when outbreaks are socially associated with a racialized group, as they have been for Africans (Ebola), Latinos (H1N1), and Asians (SARS, COVID-19) [9]. A 2021 systematic review of 16 studies found visible minorities, particularly Asian participants, are experiencing increased racial discrimination in the forms of verbal/physical abuse, hyper surveillance, and avoidance in public spaces during the current pandemic [9]. Racialized minorities have also faced more unemployment and less health care access during the pandemic compared to other groups [6]. The impacts of these experiences include increased psychological distress and reduced subjective well-being among visible minority adults and their children [10–12].

Laurencin and Walker have argued that racial discrimination and COVID-19 represent a pandemic on a pandemic that have synergized to the detriment of visible minorities and their health [13]. Across hundreds of studies and multiple systematic reviews, racial discrimination has been shown to have significant, wide-ranging adverse impacts on mental health, biological processes, and physical disease among humans [14–20]. Such profound effects are not unexpected given race-based discriminatory experiences are centered on fixed features of an individual’s physical appearance, and thus may be inherently threatening [21]. Racial discrimination is also frequently unpredictable and ongoing, offering little opportunity for individuals to place distance between themselves and the stressor to recover from its effects. Research suggests minorities rank the distress caused by discrimination as extreme, similar to the effects of major life events like the death of a loved one, divorce, and job loss [22]. Within this context, an increase in the use of psychoactive substances and other adverse coping behaviors would be expected, and a wealth of pre-pandemic studies support this contention. More than 100 studies suggest racial discrimination increases alcohol use and alcohol problems among visible minority adults [14, 23, 24]. Studies have documented links between racial discrimination and increased cigarette smoking, as well as drug use across a breadth of racial and ethnic minority groups [25–27]. Racial discrimination has also been linked to reduced engagement in health promoting behaviors such as healthcare-seeking and sleep [28–30].

In the present study, we hypothesized that racial discrimination in the early stages of the COVID-19 pandemic may have exacerbated health behavior changes already taking place among adults during this uncertain time. To examine this, we compared health behavior change among adults who did and did not experience racial discrimination during this period [31]. We also compared behavioral changes among adults who experienced racial discrimination during the pandemic to those who experienced discrimination that they did not attribute to their race. 

In summary, while many studies have shown that experiences of discrimination can result in health behavior change among adults, the extent to which discrimination may have contributed to the health behavior changes observed in the early stages of the pandemic is not well understood. The aim of this study was to examine the differential impacts of racial and non-racial discrimination on health behavior change in the first wave of the pandemic, as compared to visible minority adults who did not experience discrimination during this period.

## Methods

### Participants and Procedures

This study was approved by the University of Lethbridge Human Research Ethics Committee (ID 2020–054). All participants provided informed consent. The sample was derived from an online panel with 400,000 members that is demographically representative of the Canadian population [32]. Data collection was limited to a single province to promote consistency in terms of pandemic experiences and impacts. Alberta was nearing the end of the first wave of the pandemic during data collection, which peaked on April 30, 2020, at 68 active cases per 100,000 and fell to < 9 active cases per 100,000 by June 1, 2020, when data collection for the study began [33].

All adults within the Leger Opinion respondent pool who met eligibility criteria for this study (i.e., those who were 18 to 75 years of age and lived in Alberta, *N* = 5,552) received an email link to an online consent form. The consent form invited participants to take part in a short Qualtrics survey about their “mental, physical, and social well-being.” To reduce volunteer bias, no other details about the survey were provided. Leger Opinion supplied their standard compensation to participants who completed the survey in the form of points equivalent to about $2 CAD that can be redeemed for cash or air miles. The last page of the Qualtrics survey redirected participants to Leger Opinion to receive their compensation when they had completed the full survey.

The survey was closed when approximately 1,000 adults who opened the consent form completed and submitted the survey. Given this study had not been conducted before a sample size calculation could not be determined and the final sample size was determined on the budget available.

The recruitment and data collection process was completed in 1 week (June 1 to 7, 2020) and the final sample size was 1,050 adults.. We could not calculate a response rate for this study in the traditional sense as we did not know how many participants would have opened the link and taken part if the window to do so were left open for a longer period. Overall, 1,265 (22.8%) of the 5,552 adults who were sent the link opened it to review the consent form during the 1-week period. This included 1,050 participants who completed and submitted the survey, and 77 incompletes who began the survey but did not complete it (i.e., did not select “submit” at the end of the survey). Incompletes did not receive compensation and were not counted in the final sample size. Thus, 83% of those who opened the link for the survey completed and submitted it. Most (93%) who began the survey completed and submitted it. Participants were permitted to skip questions they did not wish to answer within the survey. Thus, 6% of participants who submitted the survey had data that were missing across one or more key variables examined in the present analysis and were removed using listwise deletion. The final sample size was *N* = 986 including 210 adults who identified with at least one non-Caucasian racial/ethnic group and 776 adults who identified as Caucasian alone.

Data for Caucasian adults could not be presented in this paper given only 2.1% reported racial discrimination in the past month (*n* = 16). This resulted in cell counts that were too small to compare against the target behavior change variables (i.e., many crosstab cell counts were under 10 participants, including some with zero counts). A greater number of Caucasian adults reported non-racial discrimination in this study; however, given non-racial discrimination was a comparator and not the central focus of this study, data for Caucasian adults were excluded.

### Measures

#### Discrimination

The 5-item Everyday Discrimination Scale (EDS) (Short Version) summed the frequency of discrimination in the day-to-day lives of adults [34, 35]. Response options are “never,” “less than once a year,” “a few times a year,” “a few times a month,” and “once a week or more.” Given our purpose was to examine discrimination during the pandemic, the 5 EDS questions were modified to ask about experiences of discrimination in the past month. To match the timeframe, EDS response options were modified to “never,” “a few times in the past month,” “once a week or more,” and “almost everyday.” The internal consistency of the modified EDS in the present sample was excellent (Cronbach’s *α* = 0.90). Given the short time frame examined, EDS scores were dichotomized to assess whether adults had experienced any discrimination in the past month (yes or no). Those who responded with “never” to each of the 5 EDS questions were coded as “no discrimination experienced in the past month.” Those who responded to at least one EDS question with “a few times in the past month” or more were coded as “yes, discrimination was experienced in the past month”.

Two additional questions asked as follows: (1) what do you think the reasons for these experiences were (select all that apply) and (2) what do you think the main reason was for these experiences (select 1). Response options for each were your race, gender, age, religion, weight, physical appearance, sexual orientation, income, mental or physical health condition or disability, people thinking you have COVID-19, and/or other. EDS responses were combined with the main attribution to create 1 variable with three categories: “no past-month discrimination,” “past-month discrimination attributed mainly to race (i.e., racial discrimination),” and “past-month discrimination attributed mainly to non-racial causes (i.e., non-racial discrimination).”

#### Increased Substance Use

Two questions asked if alcohol or cannabis had been used in the past month. If yes, participants were asked two questions to assess if their use of each substance had “increased very much,” “decreased very much,” or “stayed about the same” in the past month. Substance use was examined with abstainers included given cross-sectional studies examine prevalence and the defined population in such estimates include those at risk and not at risk for the behavior [36]. Thus, those who reported no change, decreased use, or no use in the past month were coded as “substance use did not increase in the past month.” Those who reported use had increased very much in the past month were coded as “significant substance use increase in the past month.”

#### Decreased Sleep

Participants were presented with the following statement: “In the past month, the amount that I sleep has….” Response options were “increased very much,” “decreased very much,” or “stayed about the same.” Answers were dichotomized to identify those who had decreased the amount they sleep very much (i.e., significantly) in the past month, compared to those who had not (i.e., those who did not experience a change in the amount they sleep and those who had increased the amount they sleep).

#### Decreased Exercise

Participants were presented with the following statement: “In the past month, the amount that I exercise has….” Response options were “increased very much,” “decreased very much,” or “stayed about the same.” Answers were dichotomized to identify those that had decreased their exercise very much (i.e., significantly) in the past month, compared to those who had not (i.e., those who did not change their exercise level and those who had increased it).

#### Increased Stress Eating

Participants were presented with the following statement: “In the past month, the amount of food that I stress eat has….” Response options were “increased very much,” “decreased very much,” or “stayed about the same.” Answers were dichotomized to identify those who had increased the amount they stress eat very much (i.e., significantly) in the past month, compared to those who had not (i.e., those who did not change the amount of food they ate due to stress, and those who had decreased the amount they ate due to stress).

#### Covariates

Data on age, gender, education, income, marital status, country of birth, and ethnic group were collected. Participants were also asked if they: had a positive COVID-19 test, had lost their job due to the pandemic, were an essential worker; responses to each were yes/no.

### Statistical Approach

 All adults who identified with one or more non-Caucasian racial/ethnic groups were categorized as a visible minority. This included Indigenous adults given the subsample (n = 22) was too small for a separate analysis and we did not wish to exclude them. 

Sample characteristics were examined using frequencies, crosstabs, and odds ratios (ORs). Separate logistic regression models and 95% confidence intervals (CIs) examined associations between past-month discrimination (0 = none, 1 = racial discrimination, and 2 = non-racial discrimination) and each of the 4 health behavior changes. Effect size was interpreted using the guideline that ORs greater than 2.5 are generally considered a moderate effect, and those greater than 4.0 a strong effect [37].

All models were adjusted for age, gender, education, marital status, and country of birth selected a priori based on existing literature [8]. Given job loss was common in Alberta during the pandemic, and essential workers faced working conditions that were different from other adults, models were adjusted for these covariates rather than a more standard employment variable. Covariate categories were collapsed when cell sizes were small (e.g., age was dichotomized to < 35 years and ≥ 35 years to reduce small counts when examined by discrimination). Mental health variables would be better hypothesized as mediators than confounders in this study and would require an appropriate mediation testing method (i.e., beyond simple adjustment in models) which the present sample size could not accommodate [27, 38]. All analyses were run using SPSS 27.0.

## Results

### Sample Description

Participants ranged from 18 to 75 years. No participant had received a positive COVID-19 test. This is not surprising given the pandemic was in its first wave in Alberta when data were collected and cases remained relatively low [33]. While grocery, liquor, and cannabis stores were deemed essential and remained open, most businesses had been closed and most adults were working from home during the data collection window.

About half the sample were Canadian-born (53.3%) and more than two-thirds had a post-secondary degree (Table 1). All identified with at least one non-white racial/ethnic group. The racial/ethnic makeup of the sample was similar to provincial estimates for visible minorities in Alberta with most adults identifying as Asian (54.8%), followed by Indigenous (10.5%), African American (9.0%), Middle Eastern (6.2%), Latino (4.3%), and mixed race/ethnicity (17.6%) [39]. More than a quarter of the sample (27.3%) identified as an essential worker and were going to work outside the home. Pandemic-related job loss was common in Canada at this time, and a fifth (20.6%) of visible minority adults in this sample had lost their job due to the pandemic.

**Table 1:** Sample characteristics^a^

Sample characteristics	Full sample *N* (%)	Racial discrimination subgroup, *n* (%)	Non-racial discrimination subgroup, *n* (%)
Total sample	210 (100)	56 (100)	62 (100)
Gender			
Women	99 (47.1)	24 (42.9)	30 (48.4)
Men	111 (52.9)	32 (57.1)	32 (51.6)
Age			
18–34	87 (41.4)	28 (50.0)	25 (40.3)
35–75	98 (46.7)	28 (50.0)	37 (59.7)
Post-secondary degree			
Yes	144 (68.6)	42 (75.0)	**35 (56.5)**
No	66 (31.4)	14 (25.0)	**27 (43.5)**
Married/living common-law			
Yes	123 (58.9)	30 (53.6)	35 (57.4)
No	86 (41.1)	26 (46.4)	26 (42.6)
Household income			
Low/low-middle income	77 (36.8)	17 (30.4)	28 (45.2)
Middle income	89 (42.6)	29 (51.8)	20 (32.3)
Upper-middle income	43 (20.6)	10 (17.9)	14 (22.6)
Country of birth			
Born in Canada	112 (53.3)	27 (48.2)	**42 (67.7)**
Not born in Canada	98 (46.7)	29 (51.8)	**20 (32.3)**
Essential worker			
Yes	57 (27.3)	**21 (37.5)**	17 (27.9)
No	152 (72.7)	**35 (62.5)**	44 (72.1)
Lost job due to pandemic			
Yes	43 (20.6)	10 (17.9)	16 (26.2)
No	166 (79.4)	46 (82.1)	45 (73.8)

^a^Odds ratios were used to examine differences in socioeconomic and behavior change variables by discrimination category. Cells with statistically significant differences (95% CI did not include 1) relative to no discrimination are presented in bold

### Discrimination

Most adults (56.2%) had experienced discrimination in the past month. When asked to select why (check all that apply), most attributed it partially or fully to their race (65.3%), followed by their appearance (31.4%), gender (29.7%), age (27.1%), and people believing they had COVID-19 (27.1%). Less common attributions were having a physical or mental health condition or disability (19.5%), weight (16.1%), religion (14.4%), social class (12.7%), sexual orientation (5.9%), and/or other (8.6%). Among those who experienced discrimination, the mean number of attributions was 2.7 out of 10 (*SD*: 1.8, *range* 1–9). Overall, 26.7% experienced discrimination that they attributed mainly to their race, 29.5% experienced discrimination that they attributed mainly to non-racial causes, and 43.8% did not experience discrimination in the 1-month timeframe (Table 1). Essential workers experienced more racial discrimination than other visible minority adults (38.6% vs. 25.9%; OR: 2.3, 95% CI 1.10, 4.83). Those born in Canada (OR: 2.4, 95% CI: 1.22, 4.69) and those without a post-secondary degree (OR: 1.5, 95% CI 1.04, 2.20) experienced more non-racial discrimination during the first wave of the pandemic than other visible minority adults in the sample.

Although this study was not powered to examine associations by specific racial/ethnic groups, we examined discrimination experienced by Asian participants compared to other visible minorities descriptively, given studies have documented a rise in racial discrimination targeting Asian peoples since the COVID-19 pandemic began. Overall, 31% of Asian adults in this study reported racial discrimination in the past month compared to 21% of adults from other visible minority groups. This difference was not statistically significant likely due to small cell counts. The percentage of Asian adults who reported non-racial discrimination in this timeframe was 29%, which was similar to other visible minority participants at 31%. However, when non-racial attributions were examined Asian adults were significantly more likely to attribute past-month discrimination to people believing they had COVID-19 (22%) than other visible minority adults (7%). This difference was statistically significant despite small cell counts (OR = 3.0, 95% CI: 1.35 to 6.51).

### Behavior Change

Approximately half (52.4%) of adults had made significant adverse changes in 1 or more health behaviors in the past month (*M*: 1.0 behaviors, *SD*: 1.0, *range* 0–4). A third of the sample (33.8%) had decreased exercise and 22.5% had decreased sleep. Increased stress eating was reported by 18.4%, and 9% had increased their substance use. Women were more likely to have changed at least 1 behavior (53.7%) than men (39.8%, *Chi Square*: 3.99, *p* = 0.046). There were no other significant sociodemographic differences between those who had and had not changed at least 1 health behavior in the past month.

### Discrimination and Behavior Change

Adverse health behavior change was more common among visible minority adults who had experienced discrimination in the past month (Table 2). There was a strong association between past-month racial discrimination and a significant increase in substance use (OR: 4.0), as well as a significant decrease in sleep (OR: 7.0). These associations were statistically significant but underestimated before covariate adjustment [36]. There was a small association between racial discrimination and decreased exercise (OR: 2.2) after covariate adjustment. Non-racial discrimination was strongly associated with decreased sleep, but no other health behaviors. There were no associations between either form of discrimination and changes in stress eating.

**Table 2:** Unadjusted and adjusted odds ratios (ORs) for past-month discrimination and adult health behavior change during the COVID-19 pandemic

Variables	Behavior changed (%)	Unadjusted OR (95% CI)	Adjusted OR (95% CI)^a^
Model 1: substance use increased			
No discrimination	5.4%	Reference (1.0)	Reference (1.0)
Racial discrimination	**17.9%**	**3.78 (1.22, 11.73)**	**4.04 (1.22, 13.43)**
Non-racial discrimination	6.5%	1.27 (0.33, 4.92)	1.00 (0.24, 4.11)
Model 2: sleep decreased			
No discrimination	9.8%	Reference (1.0)	Reference (1.0)
Racial discrimination	**35.7%**	**5.12 (2.13, 12.34)**	**7.00 (2.67, 18.36)**
Non-racial discrimination	**29.5%**	**3.82 (1.57, 9.32)**	**4.75 (1.80, 12.52)**
Model 3: exercise decreased			
No discrimination	29.3%	Reference (1.0)	Reference (1.0)
Racial discrimination	**42.9%**	1.81 (0.90, 3.61)	**2.18 (1.05, 4.52)**
Non-racial discrimination	32.3%	1.24 (0.61, 2.49)	1.37 (0.65, 2.86)
Model 4: stress eating increased			
No discrimination	15.4%	Reference (1.0)	Reference (1.0)
Racial discrimination	20.0%	1.38 (0.58, 3.29)	1.49 (0.60, 3.68)
Non-racial discrimination	21.3%	1.44 (0.61, 3.37)	1.37 (0.56, 3.37)

^a^Adjusted for age, gender, marital status, education, country of birth, essential worker status, and job loss due to COVID-19 using the categories presented in Table 1

## Discussion

The aim of this study was to examine the differential impacts of racial and non-racial discrimination on health behavior change among visible minority adults in the early stages of the COVID-19 pandemic. We found racial discrimination was associated with decreased sleep, increased substance use, and decreased exercise among visible minority adults during this period. This association was strongest for sleep. Decreased sleep was reported by 38% of adults who experienced past-month racial discrimination compared to 10% who had not. This translated to an odds ratio of 7 after adjustment for covariates. These findings make sense given it is well documented that racism-related vigilance is detrimental to healthy sleep [29, 40, 41]. The present results extend this literature in two ways—first by showing that the added burden of racial discrimination experienced in the context of a global stressor (an escalating pandemic) may intensify changes in sleep already taking place. Second, these findings highlight non-racial discrimination as an important determinant of reduced sleep among visible minority adults.

It is important to underline that the discrimination documented in this study took place during an especially stressful time when job loss wascommon in Canada [42]. Almost half of the adults in this study were immigrants, placing many in an even more precarious labor market position than other Canadians [43]. Approximately 1 in 5adults in this study (21%) had lost their job due to the pandemic, while 27% were essential workers during a time in which vaccines were not yet available for COVID-19. . These and other socioeconomic variables may impact health behavior change, resulting in the observed underestimate of the impact of racial and non-racial discrimination on visible minority sleep before adjustment for covariates [44].

### Discrimination and Increased Substance Use

Compared to changes in sleep, changes in substance use were less common with 9% of visible minority adults reporting that alcohol or cannabis use had increased “very much” in the pandemic’s first wave. Increased substance use was reported by approximately 18% of adults who experienced past-month racial discrimination compared to 5% who had not. This translated to an odds ratio of 4 after adjustment for covariates. These findings are consistent with research showing racial discrimination is associated with increases in substance use over time as individuals strive to cope with the psychological and physiological impacts [23, 24, 26]. Interestingly, the association between non-racial discrimination and substance use was weak and non-significant before adjustment for covariates, and reduced to an OR of 1.0 after adjustment for covariates. It may be theorized that compared to other forms of discrimination, racial discrimination is exceptionally threatening given it is based on fixed features of an individual’s physical appearance, thus resulting in a stronger coping response, including increased substance use [21, 45]. 

### Discrimination and Decreased Exercise

Little research has examined the association between racial discrimination and physical exercise, highlighting one of the unique contributions of this study to the literature. A pre-pandemic study found no association between lifetime racial discrimination and physical activity level among low-income US adults [46]. In the present study, past-month non-racial discrimination was not associated with a change in exercise habits during the early stages of the pandemic. In contrast, past-month racial discrimination was associated with a significant decrease in exercise among visible minority adults during this period (OR 2.2). While speculative, a hypothesis that could be derived from these findings is that visible minority adults who experienced racial discrimination in the early stages of the pandemic reduced or stopped exercis in public spaces in an effort to protect themselves from these experiences. This explanation is plausible given many studies and case reports have documented a marked rise in racial discrimination targeting visible minorities during the pandemic including increased threats and violence against them, and hyper surveillance and avoidance of them in public spaces, the findings of which have been widely shared in media and social media [9]. Racial discrimination can also result in a reduced sense of community belonging among ethnic minority adults, which has in turn been shown to result in more sedentary behavior [47]. Further research is needed to explore these and other potential explanations for the present findings.

Research is also recommended to explore whether racial discrimination has continued to impact the health behavior of visible minority adults in successive waves of the COVID-19 pandemic. Where possible, prospective studies with larger samples are recommended to examine both positive and negative mediators of the associations observed (e.g., sense of community belonging, mental health, allostatic load), and the downstream consequences of the behavior changes observed in this study for visible minority adults over time (e.g., substance dependence, sleep disorders). Further research is also needed to understand interventions to address and reduce discrimination, and interventions that can effectively reduce the impacts of discrimination on minority adult health [8, 20]. to help visible minority adults cope with its impacts on their health, well-being, and health behavior during and after the pandemic.

### Limitations

Study limitations include a cross-sectional design which precludes inferences about causation and the temporal sequence of variables. Although the sample was derived from a representative population, volunteer bias is a concern. A short-time window (past-month) was used to recall experiences of discrimination and changes in substance use. Despite this, recall bias and the underreporting of adverse behavior changes (i.e., social desirability bias) also remain concerns. The measure of discrimination used in this study (the EDS) is widely used and validated among visible minority adults. However, the impacts of exposure intensity (discrimination score) on health behavior change could not be explored due to the sample size. We also note that the EDS is a subjective measure designed to measure chronic, routine, and minor experiences of discrimination, and thus may not measure major experiences of discrimination [34]. The sample size also contributed to some statistical uncertainty in estimates for sleep and substance use as evidenced by wide CIs. However, the strength of the associations remains notable given the likelihood of values within a CI is maximum for the point estimate and declines as a CI moves away from it [48]. The sample size was insufficient to adjust models for individual race/ethnicity group. The sample size also could not accommodate an analysis to determine if the associations observed were consistent across different visible minority groups. Future studies with larger samples are recommended to build on the present findings through an analysis stratified by racial/ethnic group. Finally, some covariate categories were collapsed due to low cell counts which may have resulted in residual confounding.

### Conclusions

Racial discrimination had strong and significant effects on changes in substance use and sleep, and a small effect on changes in exercise during in the first wave of the pandemic. The impact of non-racial discrimination on behavior change was limited to sleep. These findings contribute to our understanding of the ways in which discrimination may intensify adverse health behavior changes among visible minority adults during a time of global crisis.

## Data Availability

The dataset on which this study was developed is available from the corresponding author on reasonable request.
